# Research on the location decision-making method of emergency medical facilities based on WSR

**DOI:** 10.1038/s41598-023-44209-0

**Published:** 2023-10-21

**Authors:** Hao Wang, Peng Luo, Yimeng Wu

**Affiliations:** 1 School of Architecture, Harbin Institute of Technology, Harbin, 150001 China; 2 Key Laboratory of Cold Region Urban and Rural Human Settlement Environment Science and Technology, Ministry of Industry and Information Technology, Harbin Institute of Technology, Harbin, 150001 China; 3 School of Design and Environment, National University of Singapore, Singapore, 117548 Singapore; 4 College of Architecture and Urban Planning, Tongji University, Shanghai, China

**Keywords:** Environmental social sciences, Natural hazards, Engineering

## Abstract

The need for emergency medical services increased drastically during disaster relief. Poor location selection of emergency medical facilities may harm the interests of healthcare workers and patients, leading to unnecessary waste of costs. It involves multiple stakeholders' interests, a typical multi-criteria decision-making problem. Based on multiple-criteria decision-making technology, most current location selection decisions methods comprehensively consider the evaluation criteria of "issue" and "problem" simultaneously and establish mathematical models to achieve the results. Such methods are difficult to take into account the influence of different attribute factors on the final location selection results in practice. Therefore, in this study, we used the WSR methodology as a guide to divide the factors of location selection into "Wuli", "Shili" and "Renli", and proposed the WSR methodology-based multi-criteria decision‐making (MCDM) framework for selecting the appropriate location for emergency medical facilities. The integrated framework consists of the Entropy Weight Method, Best–Worst Method, and interval type‐2 fuzzy Technique for Order of Preference by Similarity to Ideal Solution (TOPSIS) methodologies. Combined with the comparative analysis of actual cases, the results under the guidance of this framework were consistent with practicalities. Also, the sensitivity analysis showed that the location selection ranking fluctuations were not apparent with the fluctuation of criteria weights. Wherefore, the validation of the proposed method's effectiveness, feasibility, and robustness was proved, which provided a valuable reference for the location selection of emergency medical facilities.

## Introduction

Major emergencies, especially public health events, will have a collective health impact on large-scale populations. Also, cities often experience a surge in medical demand in a short period, leading to a shortage or even a run on regional medical resources^1–4^. From the perspective of the relationship between the supply and demand of medical resources, one solution is to build "medical evacuation points" to evacuate the wounded and reduce the medical demand in emergencies. For example, emergency medical evacuation points were built in public spaces such as the stadium and the racetrack to meet the transfer and evacuation needs of the seriously injured during the earthquake in Yushu, China^5^. Another solution is to improve the medical supplies in emergency areas to conduct "on-site treatment" by increasing temporary emergency medical facilities. For example, the United Kingdom transformed large-space public buildings such as gymnasiums and convention and exhibition centres into NHS Nightingale Hospitals in the early stage of the COVID-19 epidemic, adding additional intensive care capacity and providing vaccination services in late period^6,7^. On the whole, facing the impact of major emergencies on the traditional medical system, it is necessary to establish temporary emergency medical facilities to meet the space requirements in different rescue processes regardless of the above mitigation solutions.

Currently, the most common emergency location decision-making method is constructing a single-objective, multi-objective, or hierarchical model to perform objective calculations to get the optimal function value ^8^. The location selection of temporary emergency facilities in various regions is usually affected by specific evaluation criteria and distinct decision-making methods^9^. There are many multi-attribute decision-making methods for ranking alternative solutions, such as Technique for Order of Preference by Similarity to Ideal Solution (TOPSIS)^10^ , Multi-Attributive Border Approximation Area Comparison (MABAC)^11^, Ranking of Alternatives through Functional Single Interval (RAFSI)^12^ , Multi-Attributive Ideal-Real Comparative Assessment (MAIRCA)^13^, and Combinative Distance-based Assessment (CODAS)^14^. In specific applications, these methods possess their advantages and disadvantages. For instance, the MABAC method offers benefits such as not requiring prior weights and being intuitive and simple to comprehend. However, it may become complex when selecting sensitive data standardization and boundary construction methods. In addition, the RAFSI method can focus on the weights and relative importance between multiple attributes. However, it may have subjectivity in selecting segmentation and mapping and lacks flexibility for specific complex decision-making scenarios. Besides, the MAIRCA method can comprehensively consider the performance of multiple attributes, and its calculation is intuitive and straightforward. However, the complex weight assignment is required about the relative importance of the attributes because of the assumption that each attribute has equal weight. Moreover, although the CODAS method does not require decision-makers to assign weights, it can become computationally complex when dealing with numerous standards and alternative solutions. In public health emergencies, the site selection of temporary emergency medical facilities is often constrained by objective conditions and indirectly influenced by subjective judgments. Therefore, the decision-making process for site selection often needs to consider both "problem" and "issue" factors simultaneously. These criteria are heterogeneous and require an optimization method to assess them and their mutual influences. Considering that TOPSIS is an intuitive method for meeting the urgent needs of emergency location selection without pre-allocating weights to multiple attributes or standards and complex model construction, this paper chose the TOPSIS method for evaluation. However, its limitations lie in its dependence on weights and subjectivity in data standardization and ideal solution selection. To address the limitations, we used the WSR methodology as a guide to divide the factors of location selection into "Wuli", "Shili" and "Renli" and proposed the Multi-criteria Decision Making (MCDM) framework based on the WSR methodology. Similarly, different weight determination methods also possess their advantages and disadvantages. For example, the Ordinal Priority Approach (OPA) ^15^ allows decision-makers to allocate weights based on their preferences, meaning that weight allocation is subjective and personal bias, which may lead to inconsistent decision-making results. In addition, although the Full Consistency Method (FUCOM)^16^ can reduce the subjective influence and inconsistency of expert preferences on the final value of standard weights, its operation process is not concise because of the two sets of constraint definitions implication. Besides, The Level Based Weight Assessment (LBWA) ^17^model can eliminate inconsistencies in expert preferences with simple calculations. Still, the setting of the elasticity coefficient may affect the robustness of the final results. This article mainly adopted the Entropy Weight Method (EWM) and Best Worst Method (BWM) methods to allocate weights. EWM can make weight determination more objective and fairer without relying on the subjective preferences or weights of decision-makers; therefore, it is used as a weight determination method for "problem" factors. Besides, the BWM method is suitable for decision-making problems in various backgrounds without highly specialized mathematical or statistical knowledge and can avoid subjective bias because its weight determination is based on the relative performance of alternative solutions on various standards. Therefore, we chose the BWM method as a weight determination method for "issue" factors, though it cannot effectively handle decision-makers handling uncertainty or variability in weights. Meanwhile, this study introduced Interval Type-2 Fuzzy Sets (IT2FS) to solve weight determination's subjectivity and uncertainty problems. In emergencies, it is difficult for decision-makers to quantify their subjective cognition with crisp values in a short period, while IT2FS helps decision-makers express their preferences in a vague way, which is more closely related to the decision-making process under crises. Furthermore, facing situations of inaccurate data acquisition and incomplete understanding by decision-makers, IT2FS can represent the uncertainty range of weights through interval values. Moreover, IT2FS can make decisions more adaptable to different situations and information in emergencies to improve the robustness of decisions.

Therefore, we proposed a BWM- TOPSIS and interval type‐2 fuzzy as an integrated MCDM framework for the location selection of emergency medical facilities. The location selection decision-making framework can effectively divide different factors based on practical needs, and the primary advantage of the proposed approach is that it has simplified calculations and provides reasonable and practical solutions due to good computational efficiency compared to other methods. For validation, a case study is accompanied by Shanghai.

## Literature review

### Location selection decision

Evaluations of location selection are a challenging and complex task due to several variables which relate to specific decisions in many decision-making problems, such as environmental, social, physical, organizational, and social criteria ^18^. MCDM methods have found diverse applications across various domains, providing valuable support to decision-makers in complex decision problems^19^. Typically, since the location selection of traditional hospitals includes issues belonging to different fields and there are several and sometimes conflicting stakeholders to take into account, it chiefly uses MCDM techniques ^20^. Researchers have put forward different decision-making frameworks for hospital location selection by combining various methodologies, such as Analytical Hierarchy Process^21^, the Technique for Order Preference by Similarity to Ideal Solution, and Analytical Network Process^22^,et al. In addition, some researchers have proposed fuzzy versions of MCDM techniques based on a fuzzy mathematical calculation to consider the uncertainty and vagueness regarding the hospital location selection process. In emergency decision-making problems, especially in the COVID-19 pandemic, MCDM methods have been extensively used in taking decisions^23–26^. However, there are few studies on the location decision of emergency medical facilities in major emergencies, they follow the same technical route, mainly based on MCDM technology. For example, Nazanin Vafaei et al. proposed a combination of MCDM and GIS to meet the location selection requirements of field hospitals after the earthquake to determine their optimum location. At this stage, especially during the COVID-19 pandemic, fuzzy versions of MCDM techniques are primarily available. For example, Nezir Aydin and other researchers proposed the MCDM framework based on Delphi for selecting the most suitable location of emergency medical facilities during a pandemic, which consists of the Delphi, Best–Worst Method (BWM), and interval type‐2 fuzzy TOPSIS methodologies which is an objective DMs subjective scoring-based computational evaluation method; Muhammet and others proposed the Fuzzy Choquet integral multi criteria decision making technique for linguistic evaluation to determine the location of field hospitals during an epidemic, which eliminates subjective decision errors by using interval values for decision makers to evaluate each criterion ^27^; Chia-Nan Wang and other scholars proposed the MCDM model for the location of a temporary hospital in a fuzzy environment during the epidemic based on the fuzzy analytic hierarchy process (FAHP) and weighted aggregated sum product assessment model^28^; Ze-hui Che and others investigates an efficiency-based multi-criteria group decision-making (MCGDM) method by combining BWM and data envelopment analysis (DEA) in trapezoidal interval type-2 fuzzy (TrIT2F) environment to rank alternatives by measuring their overall efficiency^29^ .

Recently published study, some applications of BWM include identification of the best configuration of key performance indicators; fuzzy extension of the BWM and so on ^30–32^. TOPSIS‐based location selection applications include fuzzy TOPSIS to select most effective location based on interval type‐2 fuzzy TOPSIS methodologies and so on^33^. Furthermore, BWM and TOPSIS have already been applied to several MCDM problems owing to its simple and clear procedure. Hoseini used BWM and TOPSIS methods to prioritize suppliers, and they also implemented the proposed approach in type 2 fuzzy environment to deal with the uncertainty in experts' opinions^34^. Haeri developed an integrated BMW and TOPSIS approach in a fuzzy environment to prioritize suppliers^35^.

Overall, the decision-making process of the location selection methods at this stage few combine BWM with TOPSIS. And the research also rarely considers the objective evaluation criteria of "problem" and the subjective evaluation criteria of "issue" separately. However, the actual location selection decision often involves factors of different attributes in different periods, resulting in solving it difficultly in one step. The final result of the location selection decision is a staged and constantly revised process. Therefore, facing major emergencies, we need to find a new decision-making method for the location selection of emergency facilities which should be consistent with the engineering practice process. Through continuous screening and correction, the final location selection results can be achieved.

### WSR methodology

In the 1950s, the systems engineering methodology and other similar methodologies were formed to solve the organization and management of large and complex projects. Based on established work steps and thinking methods, this methodology emphasizes establishing mathematical models and quantitative analysis methods^36^. However, the reality later indicated that excessive quantification and mathematical modelling could not wholly solve specific practical issues. Faced with this problem, the International Institute for Applied Systems Analysis (IIASA) organized a seminar on the theme " Rethinking the Process of Systems Analysis". Participants concluded that quantitative methods could not be applied well to some problems, mainly due to incorrect methodologies, such as over-reliance on the establishment of mathematical models and ignoring or no explicit human factors^37^.

Based on this background, Gu Jifa and Zhu Zhichang proposed the Wuli-Shili-Renli (WSR) methodology based on engineering experience combined with traditional oriental philosophical thoughts^38^. As a systematic tool for solving complex problems, the methodology decomposes the complexities into three interconnected dimensions: Wuli, Shili, and Renli. Wuli refers to the objective of the existence of things and laws, including the physical environment and structural organization, etc., as principles and rules for dealing with specific affairs in the real world. "Shili" emphasizes the mode of interaction between people and the "world", which is an intervention or processing mechanism. "Renli" emphasizes the subjective relationship between all parties involved in systems engineering, especially the subjective role of people.

Since the WSR methodology is appropriate for dealing with complex issues in a hierarchical and organized manner by reasonably coordinating the complex connections between various factors, scholars in different fields have begun to use this methodology to research complex issues. Some researchers use the WSR method to construct evaluation systems, such as the government credit evaluation index system ^39^and the comprehensive index system of sustainability evaluation of the shale gas industry^40^. In addition, some researchers also use the WSR method to build theoretical models. For example, Abdelbasset W K and others proposed a service model based on WSR theory and contributed to the theoretical research of delivery services . Lin and others analyzed the complexity of knowledge management and established a general knowledge management model for the Knowledge Management System^41^ . Furthermore, other researchers combined the WSR method with other models or theories and conducted quantitative calculations and demonstrations. For example, Gen Li and others determined the energy intensity factor system of the manufacturing industry based on the WSR method. They used the VAR model to analyze various factors influencing the energy intensity of the manufacturing industry ^42^. Based on the WSR methodology, Jinxian Zhao and others introduced the fuzzy theory to develop a comprehensive subway shield construction evaluation model based on combined weighting by the multiplicative synthesis method ^43^.

The above analysis shows that the WSR method is appropriate for dealing with complex systems and things. Through dividing various factors into “Wuli”, “Shili” and “Renli” according to their attributes, WSR method provides different solutions under the constraints of various factors. The location selection of emergency medical facilities is a complex problem of multi-factor interaction and influence, so the WSR method derived from engineering practice experience can be used to decompose this complex problem. By dismantling complex problems into different solution stages according to the different attributes of the influencing factors, each stage can meet specific needs to obtain the optional result. Compared with the previous simple application of multi-objective optimization model or MCDM method for one-shot modeling solution, this hierarchical location decision-making idea is more in line with the operation process of engineering practice, and it can also more conveniently consider the effects of different attribute factors on the location decision-making.

## Method

### Location selection decision workflow

**Figure 1:**
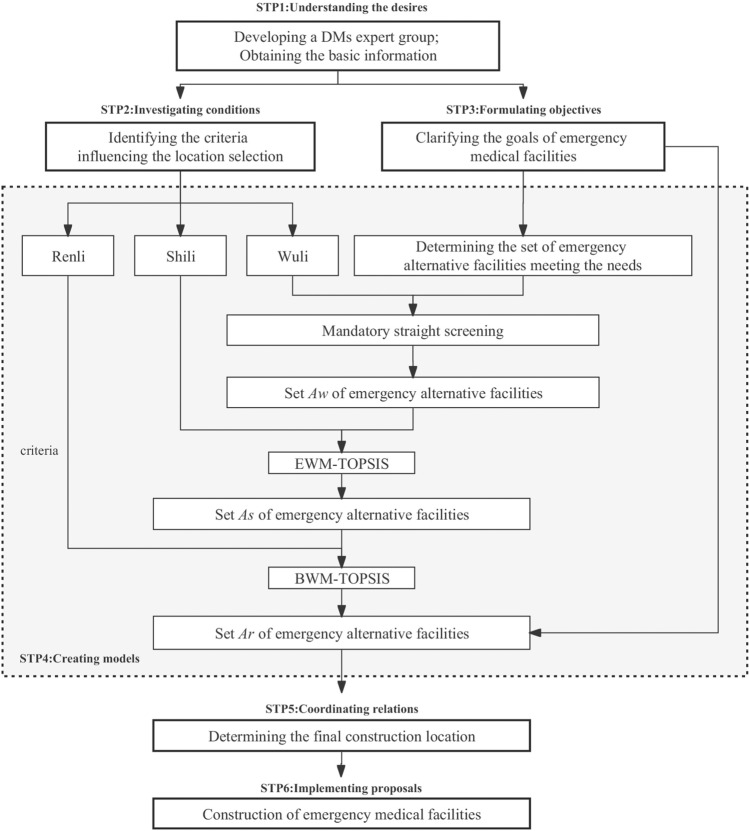
Location diagram of the alternative facilities.

The WSR methodology recommends six elements of action: understanding desires, investigating conditions, formulating objectives, creating models, coordinating relations and implementing proposals. This study develops an MCDM method based on the WSR methodology (Fig. 1). The specific steps of the integrated method are detailed as follows:

Step 1: Understanding desires: A DMs expert group is developed and the actual situation in the current region and basic information such as the type and level of emergencies are obtained;

Step 2: Investigating conditions: According to the specific situation of the current area and emergency, the set of factors *C* is identified that affect the location selection under such emergencies, and the relevant factors are divided into "Wuli" factors ${C}_{w}$=$({c}_{w1,}{c}_{w2,}\dots ,{c}_{wn,}$), the “Shili” factor ${C}_{s}$=$({c}_{s1,}{c}_{s2,}\dots ,{c}_{sm,}$) and the “Renli” factor Factors ${C}_{r}$=$({c}_{r1,}{c}_{r2,}\dots ,{c}_{rt,}$) three categories. At the same time, the actual data required by factors ${C}_{s},{C}_{r}$ and ${C}_{w}$ is collected;

Step 3: Formulating objectives: Potential alternative facility set $A$ and the construction type and quantity of emergency medical facilities according to the actual situation are determined;

Step 4: Creating models: A screening model for alternative facilities is developed, and the order of action of the "Wuli" factor, "Shili" factor and "Renli" factor is clarified. The alternative locations that do not meet objectivity requirements from the "Wuli" factor should be directly excluded. The "Shili" factor is an artificially formulated and quantifiable evaluation standard based on the actual situation, whose weight can be given by the entropy weight method objectively, and then the factors would be sorted by the TOPSIS method. The "Renli" factors refer to the evaluation criteria for subjective judgments based on decision-makers experience. The BWM method calculates subjective weights to the "Renli" factors, and the remaining alternative facilities screened by the "Shili" factors are ranked by applying the interval type-2 fuzzy TOPSIS method;

Step 5: Coordinating relations: According to the actual situation of the city, which alternative facilities can be used to build emergency medical facilities can be finalized after the screening of the fourth step model;

Step 6: Implementing proposals: Construction can begin according to the location decided by the city's final decision maker.

### Mathematical models

Step 4, "Creating models", is the core link in the above workflow framework, which is one of the crucial innovations of this study. The screening models of alternative facilities would directly influence the final decision result. The specific steps and mathematical models are as follows:

Step 4.1: According to the actual situation, the DMs expert group need to decide the order of action of "Wuli", "Shili" and "Renli" factors. The following will explain the selection process of alternative facilities in the order of "Wuli"-"Shili"-"Renli".

Step 4.2: The "Wuli" factor is an objective requirement. Compared the obtained actual data of the evaluation index with the required numerical value of the index according to the "Wuli" factor ${C}_{w}$, the candidate locations that do not meet the "wuli" factor are directly excluded from forming the candidate location ${A}_{w}$;

Step 4.3: The "Shili" factor is human intervention. At this time, the "Shili" factor should be objectively weighted by the entropy weight method, and the TOPSIS method should be used for sorting and screening. The specific process is as follows:

Step 4.3.1: Constructing the initial matrix. Firstly, the mathematical model of each factor is established according to the "Shili" factor ${C}_{s}$. Then, combined with the candidate location ${A}_{w}$, the initial evaluation matrix $B=({b}_{ij}{)}_{m\times n}$ is formed according to the objective calculation results of each factor, where ${b}_{ij}$ is the evaluation index ($i=\mathrm{1,2},3,\dots$, $m$; $j$=$\mathrm{1,2},3,\dots$, $n$), indicating the numerical value of the $j$ th evaluation index in the $i$ th evaluation object.

$$B=\left[\begin{array}{cccc}{b}_{11}& {b}_{12}& ...& {b}_{1n}\\ {b}_{21}& {b}_{22}& ...& {b}_{2n}\\ ...& ...& ...& ...\\ {b}_{m1}& {b}_{m2}& ...& {b}_{mn}\end{array}\right]$$

Step 4.3.2: Normalizing the initial matrix. The dimensions of each index factor are different in the "Shili" evaluation index, which lead to the impossibility of direct comparison and comprehensive evaluation. In order to eliminate the influence of the dimension on the evaluation results, it is necessary to perform dimensionless processing on the index by establishing a standardized decision matrix $F=({f}_{ij}{)}_{m\times n}$, ${f}_{ij}$ represents the $j$ th evaluation index value in the $i$ th evaluation object after normalization ($i=\mathrm{1,2},3,\dots$, $m$; $j$=$\mathrm{1,2},3,\dots$, $n$).

$${\mathrm{For\, positive \,indicators}: f}_{ij}=\frac{{b}_{ij}-{b}_{j}^{min}}{{b}_{j}^{max}-{b}_{j}^{min}}$$

$${\mathrm{For \,negative\, indicators}: f}_{ij}=\frac{{{b}_{j}^{max}-b}_{ij}}{{b}_{j}^{max}-{b}_{j}^{min}}$$

Step 4.3.3: Determining the weight of each evaluation index by entropy weight method. The index weight $${w}_{j} (j=\mathrm{1,2},\begin{array}{c}...\end{array},n$$)in the "factual" factor ${C}_{s}$ is determined by using the entropy weight method.

Step 4.3.4: Weighting the normalized matrix. The normalized decision matrix F is weighted to form the matrix $H=({h}_{ij}{)}_{m\times n}$, ${h}_{ij}$ represents the $i$ th weighted normalized evaluation value in the $j$ th evaluation object, ${h}_{ij}$ = ${w}_{j}\times {f}_{ij}$ ($i=\mathrm{1,2},3,\dots$, $m$; $j$ = $\mathrm{1,2},3,\dots$, $n$).

Step 4.3.5: Determining the positive ideal solution ${h}_{j}^{+}$ and the negative ideal solution ${h}_{j}^{-}$, and calculating the distance from each evaluation object to the positive ideal solution ${d}_{j}^{+}$ and the negative ideal solution ${d}_{j}^{-}$ ($i=\mathrm{1,2},3,\dots$, $m$; $j$ = $\mathrm{1,2},3,\dots$, $n$).

$${d}_{j}^{+}=\sqrt{\sum_{j=1}^{n}({h}_{ij}{-{h}_{j}^{+})}^{2}}$$

$${d}_{j}^{-}=\sqrt{\sum_{j=1}^{n}({h}_{ij}{-{h}_{j}^{-})}^{2}}$$

$${h}_{j}^{+}=max({h}_{ij})$$

$${h}_{j}^{-}=min({h}_{ij})$$

Step 4.3.6: Calculating the closeness ${E}_{i}$ of the alternative location ${A}_{w}$ and idealized targets.

$${E}_{i}=\frac{{d}_{j}^{-}}{{d}_{j}^{+}+{d}_{j}^{-}}$$

Step 4.3.7: According to the closeness ${E}_{i}$, selecting from the alternative location ${A}_{w}$ to form the alternative location ${A}_{s}$.

Step 4.4: The "Renli" factor ${C}_{r}$ is the subjective evaluation of human beings. BWM and TOPSIS method are used to screen facilities ${A}_{s}$ and form the final set of alternative facilities ${A}_{r}$ according to the evaluation of DMs. Type‐2 fuzzy sets are an extension of the fuzzy set theory proposed by Zade, which is mainly focus on dealing with uncertainty to produce more accurate and robust results^44–46^. The Mathematical calculation rules of the type‐2 fuzzy set have also been sufficient in the relevant literatures which is beneficial to facilitate calculations ^47–49^.

Step 4.4.1: Determining the criteria ${C}_{r}$ weight by the BWM method. Once the DMs evaluated criteria ${C}_{r}$ using the values 1–9, the BWM is applied to determine the criteria weights $${w}_{z} (z=\mathrm{1,2},\begin{array}{c}...\end{array},\widetilde{n})$$ of the "Renli" factors.

Step 4.4.2: Constructing the initial matrix. According to the "Renli" factor ${C}_{r}$ index, DMs subjectively evaluate the alternative location ${A}_{s}$ after the "reasonable" factor screening, and convert the linguistic terms into a type-2 fuzzy set ^50^to form an initialization evaluation matrix $P=(\widetilde{{p}_{\tilde{i} \tilde{j}}}{)}_{m\times n}$. $\widetilde{{p}_{\tilde{i} \tilde{j}}}$ is the evaluation index, indicating the numerical value of the $\widetilde{i}$ evaluation index in the $\widetilde{j}$ evaluation object ($\widetilde{i}=\mathrm{1,2},3,\dots$, $\widetilde{m}$; $\widetilde{j}$ = $\mathrm{1,2},3,\dots$, $\widetilde{n}$). The following linguistic terms (Table 1) are used in DMs assessments of the alternatives considering the determined criteria.

**Table 1:** Linguistic terms to evaluate the alternatives.

Linguistic terms	Interval type‐2 fuzzy sets
Very low (VL)	((0, 1, 1, 3; 1, 1), (0.5, 1, 1, 2; 0.9, 0.9))
Low (L)	((1, 3, 3, 5; 1, 1), (2, 3, 3, 4; 0.9, 0.9))
Medium low (ML)	((3, 5, 5, 7; 1, 1), (4, 5, 5, 6; 0.9, 0.9))
Medium (M)	((5, 7, 7, 9; 1, 1), (6, 7, 7, 8; 0.9, 0.9))
Medium high (MH)	((7, 9, 9, 10; 1, 1), (8, 9, 9, 9.5; 0.9, 0.9))
High (H)	((8, 9, 10, 10; 1, 1), (9, 9, 9, 10; 0.9, 0.9))
Very high (VH)	((9, 10, 10, 10; 1, 1), (9.5, 10, 10, 10; 0.9, 0.9))

$$P=(\widetilde{{p}_{\tilde{i} \tilde{j}}})=\left[\begin{array}{cccc}\widetilde{{p}_{11}}& \widetilde{{p}_{12}}& ...& \widetilde{{p}_{1n}}\\ \widetilde{{p}_{21}}& \widetilde{{p}_{22}}& ...& \widetilde{{p}_{2n}}\\ ...& ...& ...& ...\\ \widetilde{{p}_{m1}}& \widetilde{{p}_{m2}}& ...& \widetilde{{p}_{mn}}\end{array}\right]$$

where $$\widetilde{{p}_{\tilde{i} \tilde{j}}}$$ = (($${a}_{11}^{u}$$, $${a}_{12}^{u}$$, $${a}_{13}^{u}$$, $${a}_{14}^{u}$$; $${H}_{1}({A}_{1}^{u})$$, $${H}_{2}({A}_{1}^{u})$$), ($${a}_{11}^{l}$$, $${a}_{12}^{l}$$, $${a}_{13}^{l}$$, $${a}_{14}^{l}$$; $${H}_{1}({A}_{1}^{l})$$, $${H}_{2}({A}_{1}^{l})$$).

Step 4.4.3: Constructing the normalized Initial matrix. In the "Renli" evaluation factor, the normalization process is performed to convert multiple criteria measures into similar measures to establish a standardized decision matrix $R$, so as to eliminate the influence of different indicators on the evaluation results. $\widetilde{{r}_{\tilde{i} \tilde{j}}}$ refers to the numerical value of the $\widetilde{j}$ evaluation index in the $\widetilde{i}$ evaluation object after normalization ($\widetilde{i}=\mathrm{1,2},3,\dots$, $\widetilde{m}$; $\widetilde{j}$=$\mathrm{1,2},3,\dots$, $\widetilde{n}$).

$$R=(\widetilde{{r}_{\tilde{i} \tilde{j}}})=\left[\begin{array}{cccc}\widetilde{{r}_{11}}& \widetilde{{r}_{12}}& ...& \widetilde{{r}_{1n}}\\ \widetilde{{r}_{21}}& \widetilde{{r}_{22}}& ...& \widetilde{{r}_{2n}}\\ ...& ...& ...& ...\\ \widetilde{{r}_{m1}}& \widetilde{{r}_{m2}}& ...& \widetilde{{r}_{mn}}\end{array}\right]$$

For positive indicators:

$$\widetilde{{r}_{\tilde{i} \tilde{j}}}=\left(\left(\frac{{a}_{\widetilde{i}1}^{u}}{{a}_{\widetilde{i}*}^{u}},\frac{{a}_{\widetilde{i}2}^{u}}{{a}_{\widetilde{i}*}^{u}},\frac{{a}_{\widetilde{i}3}^{u}}{{a}_{\widetilde{i}*}^{u}},\frac{{a}_{\widetilde{i}4}^{u}}{{a}_{\widetilde{i}*}^{u}}; {H}_{1}({A}_{\widetilde{j}}^{u}), {H}_{2}({A}_{\widetilde{j}}^{u}\right)),\left(\frac{{a}_{\widetilde{i}1}^{l}}{{a}_{\widetilde{i}*}^{u}},\frac{{a}_{\widetilde{i}2}^{l}}{{a}_{\widetilde{i}*}^{u}},\frac{{a}_{\widetilde{i}3}^{l}}{{a}_{\widetilde{i}*}^{u}},\frac{{a}_{\widetilde{i}4}^{l}}{{a}_{\widetilde{i}*}^{u}}{; H}_{1}({A}_{\widetilde{i}}^{u}){, H}_{2}({A}_{\widetilde{i}}^{l})\right)\right)$$

For negative indicators:

$$\widetilde{{r}_{\tilde{i} \tilde{j}}=}\left(\left(\frac{{a}_{{\widetilde{i}}^{-}}^{u}}{{a}_{\widetilde{i}4}^{u}},\frac{{a}_{{\widetilde{i}}^{-}}^{u}}{{a}_{\widetilde{i}3}^{u}},\frac{{a}_{{\widetilde{i}}^{-}}^{u}}{{a}_{\widetilde{i}2}^{u}},\frac{{a}_{{\widetilde{i}}^{-}}^{u}}{{a}_{\widetilde{i}1}^{u}}{; H}_{1}({A}_{\widetilde{i}}^{u}){, H}_{2}({A}_{\widetilde{i}}^{u})\right),\left(\frac{{a}_{{\widetilde{i}}^{-}}^{u}}{{a}_{\widetilde{i}4}^{l}},\frac{{a}_{{\widetilde{i}}^{-}}^{u}}{{a}_{\widetilde{i}3}^{l}},\frac{{a}_{{\widetilde{i}}^{-}}^{u}}{{a}_{\widetilde{i}2}^{l}},\frac{{a}_{{\widetilde{i}}^{-}}^{u}}{{a}_{\widetilde{i}1}^{l}}; {H}_{1}({A}_{\widetilde{i}}^{l}){, H}_{2}({A}_{\widetilde{i}}^{l})\right)\right)$$

$${a}_{\widetilde{i}*}^{u}=\underset{\widetilde{i}}{max}{ a}_{\tilde{i} \tilde{j}}^{u}\widetilde{ j}\in\, B\, B\, \mathrm{are\, the\, sets \,of\,benefit\, criteria}$$

$${a}_{{\widetilde{i}}^{-}}^{u}=\underset{\widetilde{i}}{min}{ a}_{\tilde{i} \tilde{j}}^{u}\widetilde{ j}\in\, C \,C\, \mathrm{are \,the\, sets \,of \,cost \,criteria}$$

Step 4.4.4: Weighting the normalized matrix. The normalized decision matrix $R$ is given weight to form a decision matrix $V=\left(\widetilde{{v}_{\tilde{i} \tilde{j}}}\right)_{m\times n}$. $\widetilde{{v}_{\tilde{i} \tilde{j}}}$ refers to the $\widetilde{j}$ th weighted normalized evaluation numerical value in the $\widetilde{i}$th evaluation object ($\widetilde{i}=\mathrm{1,2},3,\dots$, $\widetilde{m}$; $\widetilde{j}$=$\mathrm{1,2},3,\dots$, $\widetilde{n}$).

$$\widetilde{{v}_{\tilde{i} \tilde{j}}}={w}_{z}\times \widetilde{{r}_{\tilde{i} \tilde{j}}}$$

Step 4.4.5: Determining the positive ideal solution ${v}_{\widetilde{j}}^{+}$ and the negative ideal solution ${v}_{\widetilde{j}}^{-}$, and calculating the distance from each evaluation object to the positive ideal solution ${d}_{\widetilde{i}}^{+}$ / the negative ideal solution ${d}_{\widetilde{i}}^{-}$ according to the $Rank(\widetilde{{v}_{\tilde{i} \tilde{j}})}$ ^51^and ${v}_{\widetilde{j}}^{+}$/${v}_{\widetilde{j}}^{-}$ .

$${d}_{\widetilde{i}}^{+}=\sqrt{\sum_{j=1}^{m}(Rank(\widetilde{{v}_{\tilde{i} \tilde{j}})}{-{v}_{\widetilde{j}}^{+})}^{2}}$$

$${d}_{\widetilde{i}}^{-}=\sqrt{\sum_{j=1}^{m}(Rank(\widetilde{{v}_{\tilde{i} \tilde{j}})}{-{v}_{\widetilde{j}}^{-})}^{2}}$$

$$\mathrm{Rank}(\widetilde{{v}_{\tilde{\mathrm{i}} \tilde{\mathrm{j}}})}={\mathrm{M}}_{1}({v}_{\tilde{\mathrm{i}} \tilde{\mathrm{j}}}^{\rm{u}})$$+ $${\mathrm{M}}_{1}({v}_{\tilde{\mathrm{i}} \tilde{\mathrm{j}}}^{\rm{l}})$$+ $${\mathrm{M}}_{2}({v}_{\tilde{\mathrm{i}} \tilde{\mathrm{j}}}^{\rm{u}})$$+ $${\mathrm{M}}_{2}({v}_{\tilde{\mathrm{i}} \tilde{\mathrm{j}}}^{\rm{l}})$$+ $${\mathrm{M}}_{3}({v}_{\tilde{\mathrm{i}} \tilde{\mathrm{j}}}^{\rm{u}})$$+ $${\mathrm{M}}_{3}({v}_{\tilde{\mathrm{i}} \tilde{\mathrm{j}}}^{\rm{l}}) -\frac{1}{4} ({\mathrm{S}}_{1}({v}_{\tilde{\mathrm{i}} \tilde{\mathrm{j}}}^{\rm{u}})$$+ $${\mathrm{S}}_{1}({v}_{\tilde{\mathrm{i}} \tilde{\mathrm{j}}}^{\rm{l}})$$+ $${\mathrm{S}}_{2}({v}_{\tilde{\mathrm{i}} \tilde{\mathrm{j}}}^{\rm{u}})$$+ $${\mathrm{S}}_{2}({v}_{\tilde{\mathrm{i}} \tilde{\mathrm{j}}}^{\rm{l}})$$+ $${\mathrm{S}}_{3}({v}_{\tilde{\mathrm{i}} \tilde{\mathrm{j}}}^{\rm{u}})$$+ $${\mathrm{S}}_{3}({v}_{\tilde{\mathrm{i}} \tilde{\mathrm{j}}}^{\rm{l}})$$+ +$${\mathrm{S}}_{4}({v}_{\tilde{\mathrm{i}} \tilde{\mathrm{j}}}^{\rm{u}}){\mathrm{S}}_{4}({v}_{\tilde{\mathrm{i}} \tilde{\mathrm{j}}}^{\rm{l}})$$)) + $${\mathrm{H}}_{1}({v}_{\tilde{\mathrm{i}} \tilde{\mathrm{j}}}^{\rm{u}})$$+ $${\mathrm{H}}_{1}({v}_{\tilde{\mathrm{i}} \tilde{\mathrm{j}}}^{\rm{l}})$$+ $${\mathrm{H}}_{2}({v}_{\tilde{\mathrm{i}} \tilde{\mathrm{j}}}^{\rm{u}})$$+ $${\mathrm{H}}_{2}({v}_{\tilde{\mathrm{i}} \tilde{\mathrm{j}}}^{\rm{l}}).$$

Among $${\mathrm{M}}_{\rm{p}}({v}_{\tilde{\mathrm{i}} \tilde{\mathrm{j}}}^{N})$$=(($${v}_{\tilde{\mathrm{i}} \tilde{\mathrm{p}}}^{N}$$+$${v}_{\widetilde{\mathrm{i}}\widetilde{(\mathrm{p}+1)}}^{N}$$)/2)

$${\mathrm{H}}_{\rm{w}}\left({v}_{\tilde{\mathrm{i}} \tilde{\mathrm{j}}}^{N}\right)={v}_{\widetilde{\mathrm{i}}\widetilde{(\mathrm{w}+1)}}^{N}$$

$${\mathrm{S}}_{\rm{q}}\left({v}_{\tilde{\mathrm{i}} \tilde{\mathrm{j}}}^{N}\right)=\sqrt{\frac{1}{2}\sum_{\rm{q}}^{\rm{q}+1}({v}_{\tilde{\mathrm{i}} \tilde{\mathrm{p}}}^{N}{-\frac{1}{2}\sum_{\rm{q}}^{\rm{q}+1}{v}_{\tilde{\mathrm{i}} \tilde{\mathrm{p}}}^{N})}^{2}}$$

$${\mathrm{S}}_{4}\left({v}_{\tilde{\mathrm{i}} \tilde{\mathrm{j}}}^{\rm{N}}\right)=\sqrt{\frac{1}{4}\sum_{1}^{4}({v}_{\tilde{\mathrm{i}} \tilde{\mathrm{p}}}^{N}{-\frac{1}{4}\sum_{1}^{4}{v}_{\tilde{\mathrm{i}} \tilde{\mathrm{p}}}^{N})}^{2}}$$

$$1\le p\le 31\le q\le 3; 1\le w\le 2$$

$$N$$∈{$$u$$,$$l$$}; $$\widetilde{i}=\mathrm{1,2},3,\dots $$, $$\widetilde{m}$$; $$\widetilde{j}$$=$$\mathrm{1,2},3,\dots $$, $$\widetilde{n}$$

Step 4.4.6: Calculating the closeness of the alternative location ${A}_{w}$ and the idealized targets $\widetilde{{E}_{\widetilde{i}}}$.

$$\widetilde{{E}_{\widetilde{i}}}=\frac{{v}_{\widetilde{j}}^{-}}{{v}_{\widetilde{j}}^{-}+{v}_{\widetilde{j}}^{+}}$$

Step 4.4.7: Ranking the alternative facilities ${A}_{s}$ considering the closeness $\widetilde{{E}_{\widetilde{i}}}$, so to form the final set of alternative facilities ${A}_{r}$.

## Application

Facing excessive medical demand load caused by major emergencies, it is difficult for the government to guarantee to host and treat their patients in fully equipped hospitals. In emergency rescue, public buildings such as gymnasiums and convention and exhibition centres have the strengths of large spatial scale, high security and so on^52^. They have served as separate emergency medical facilities in various types of disaster relief operations in different countries, such as the Fangcang shelter hospital ^53^, the NHS Nightingale Hospital in public health emergencies and the alternative care facility ^54–56^ and the medical evacuation point during natural disasters. Previous cases have fully demonstrated that large-space public buildings represented by gymnasiums can supplement the need for beds in the healthcare system and decompose the system pressure quickly when the urban medical system is facing collapse. In addition, emergency medical facilities transformed from large-space public buildings are easy to construct and will not cause too much social and economic burden. However, one of the most critical problems is how to screen the optimal from numerous large public buildings which are the potential alternative locations.

As emergency medical facilities, the Fangcang shelter hospitals' location are selected from the large-space public buildings such as gymnasiums and convention and exhibition centres to serve patients who are infected with COVID‐19. Therefore, this paper applies the proposed emergency location decision method to the selection of the location of the Fangcang shelter hospitals during public health emergencies.

### Problem description

As an international metropolis and one of the largest commercial centers in the world, Shanghai has the high human circulation and is vulnerable to the impact of the epidemic. In March 2022, the COVID-19 epidemic in Shanghai had a major impact on the medical system. Shanghai launched the construction of Fangcang shelter hospitals to treat patients with mild symptoms to ensure the safety of medical supplies and the health of urban residents. 

**Figure 2:**
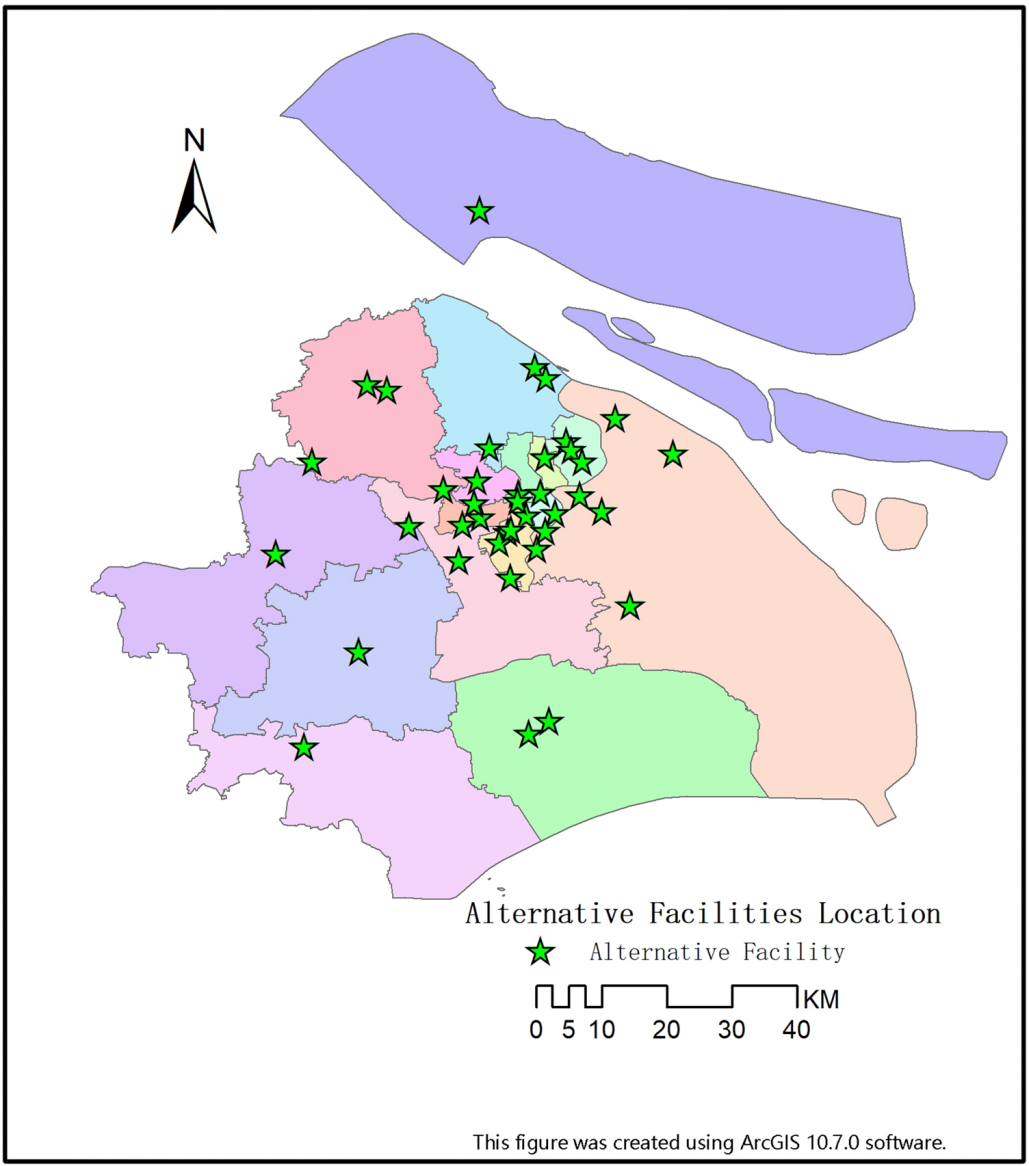
Alternative facilities location.

In response to this public health emergency, we selected the available within the city as alternative facility points set $A$ which contains 39 large-space public buildings (Fig. 2), including 29 gymnasiums and 10 convention and exhibition centres. The government need to develop a DMs expert group based on the proposed location selection decision-making method to fully understand the epidemic information and related needs. Meantime, combined with the actual situation and relevant specifications of respiratory infectious diseases, 8 criteria affecting the location selection of Fangcang shelter hospitals were identified through expert discussion and the selection criteria were explained as follows:

### A greening distance of at least 20 m should be reserved between Fangcang shelter hospitals and the surroundings (C1)

The selected locations of the Fangcang shelter hospitals must keep a safe distance from the surrounding environment, for avoiding potential impact on the surroundings.

### Distance to hospital (C2)

**Figure 3:**
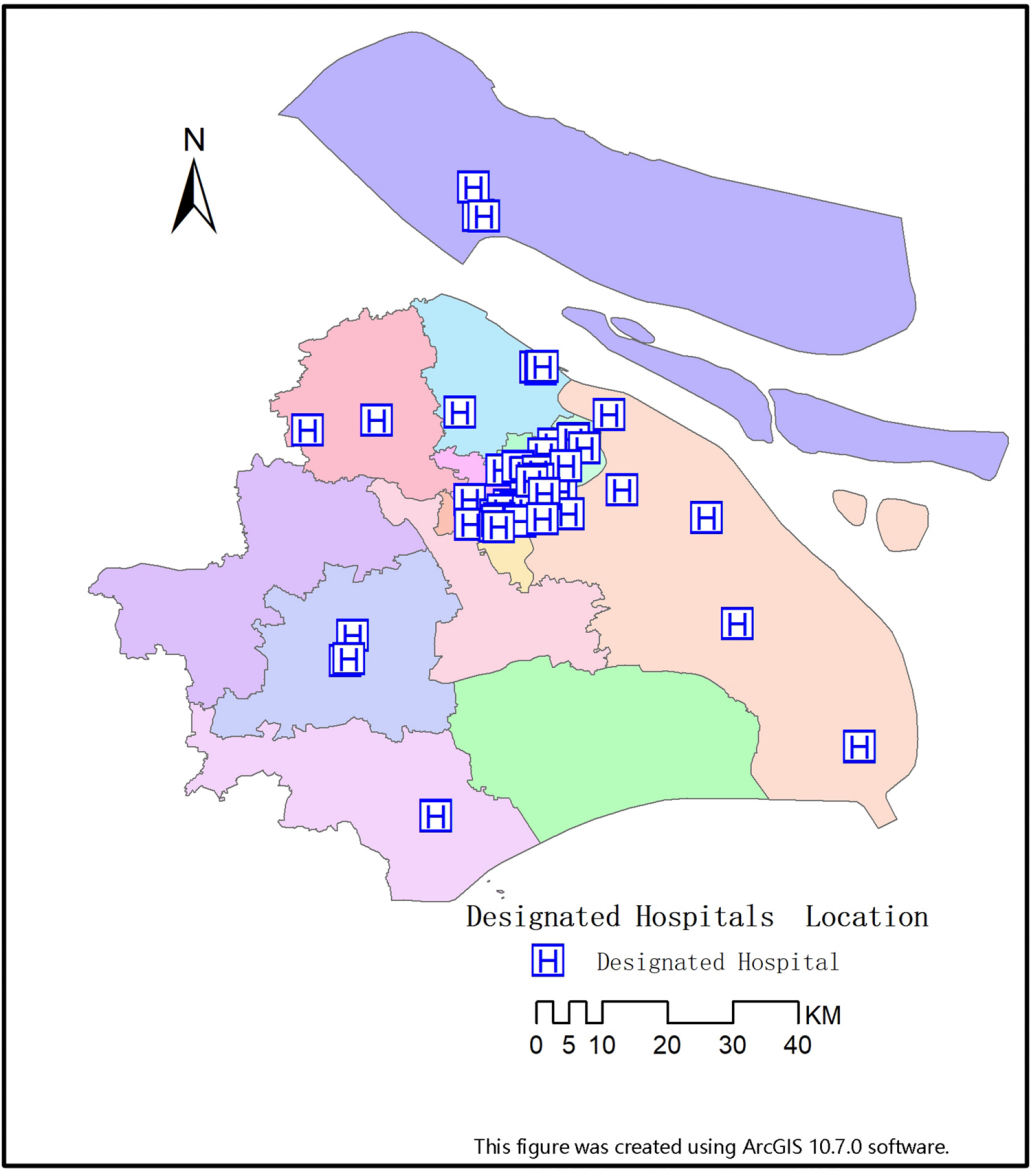
Designated hospitals location.

Large‐scale areas being evaluated should be close to an infectious disease hospital or third-grade class-A hospital so that the patients whose illness worsen from mild to severe can be rapidly transferred for further treatment (Fig. 3).

### Spatial scale (C3)

In case of emergency, Fangcang shelter hospitals should choose Large‐scale buildings with sufficient indoor space for construction which can be expanded with additional bed capacity in a short time.

### Accessibility (C4)

**Figure 4:**
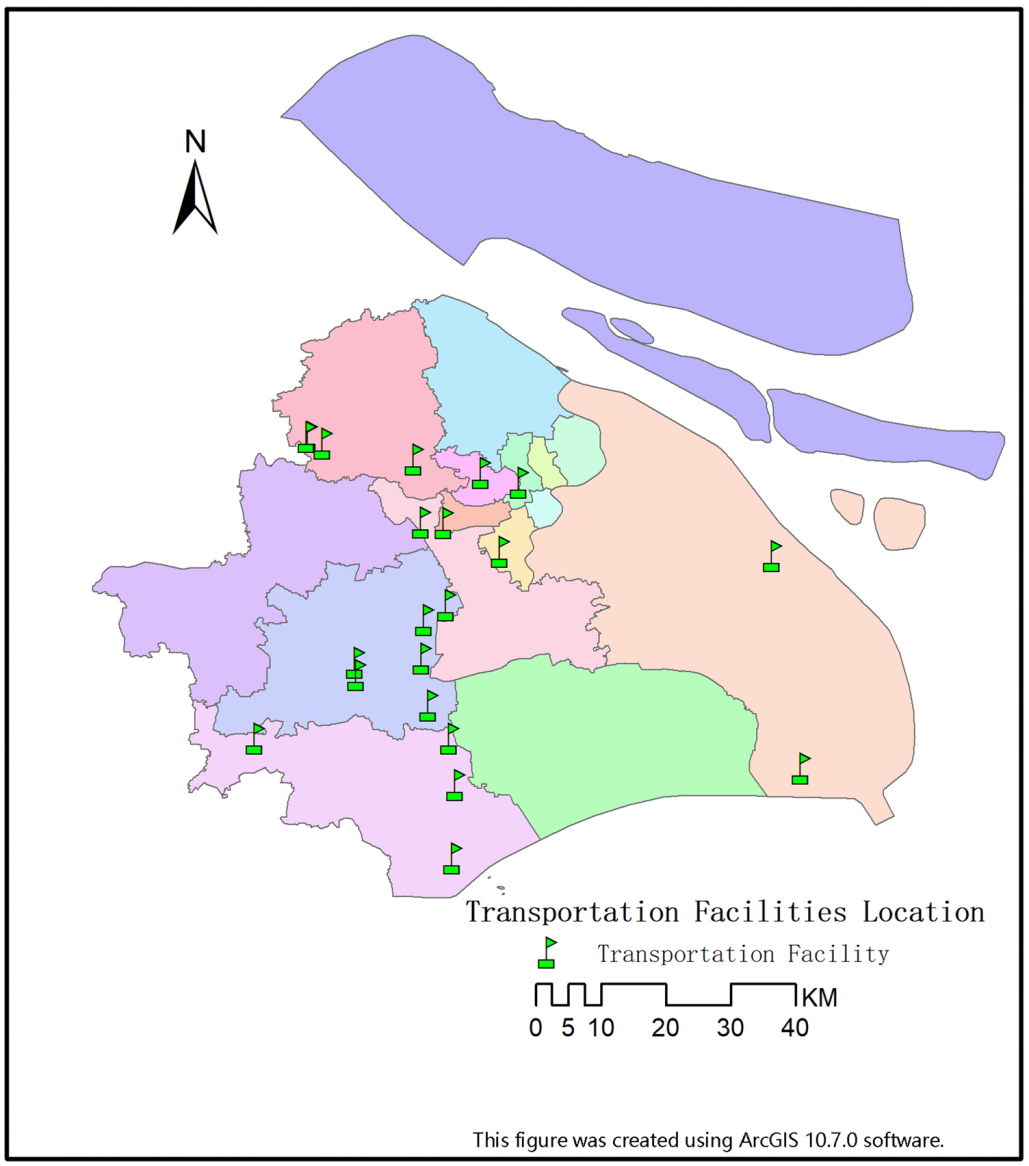
Transportation facilities location.

**Figure 5:**
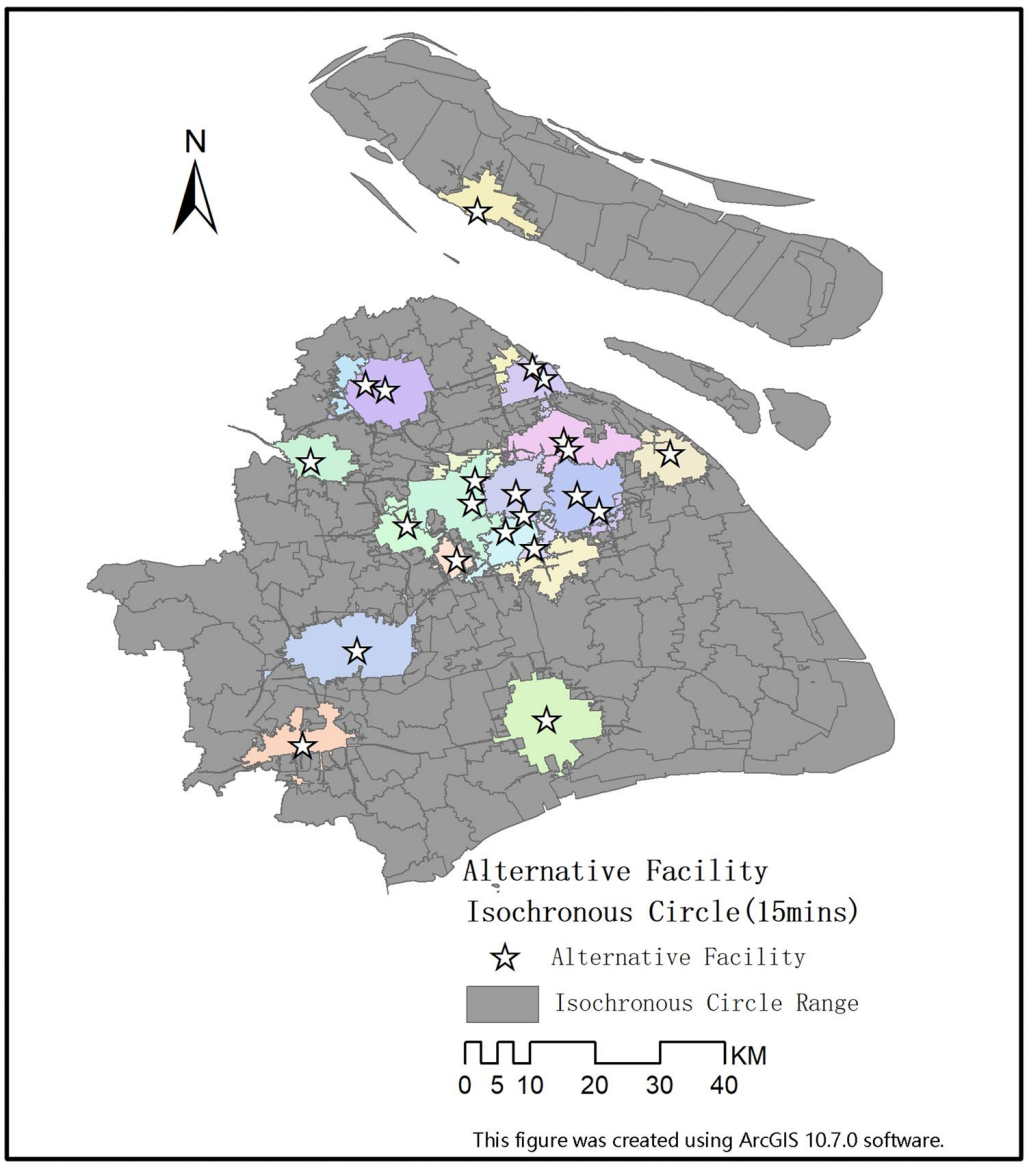
Alternative facilities isochronous circle (15 min).

The location selection of Fangcang shelter hospitals not only needs to take into account the surrounding road conditions, but also the connection with the transportation hub. Because it is necessary to facilitate the arrival of surrounding patients, as well as the arrival of external aid materials and personnel (Figs. 4, 5).

### Post-pandemic building recovery (C5)

Consideration should be given to the difficulty of restoring the original function. Because expropriated buildings need to restore their original functions after the epidemic.

### Operability (C6)

The degree of matching between the requisitioned building space and the function of the Fangcang shelter hospitals affects the difficulty of construction.

### Implementation efficiency (C7)

Consideration should be given to the time cost of completing the reconstruction of the requisitioned building, because the construction of the Fangcang shelter hospitals should be completed in a short time.

### Operational effect (C8)

Since Fangcang shelter hospitals separate patients from their relatives and society, enough space should be available to socialize and receive various types of services,such as medical care, monitoring, nutrition, accommodation.

The DMs expert group divided the eight impact criteria into the factors of "Wuli", "Shili" and "Renli" (Table 2), and decided to screen the candidate points in the order of "Wuli"–"Shili"–"Renli" principles according to the actual situation.

**Table 2:** Classification and composition of location selection criteria for Fangcang shelter hospitals.

Classification	Criteria composition of location selection
"Wuli"	A greening distance of at least 20 m should be reserved between Fangcang shelter hospital and the surroundings
"Shili"	Accessibility
	Distance to hospital
	Spatial scale
"Renli"	Operability
	Post-pandemic building recovery
	Operational effect
	Implementation efficiency

### Application procedure

The evaluation index of the "Wuli" factor often has precise objective requirements. The construction guidelines of the Fangcang shelter hospital require that a greening distance of at least 20 m be reserved between it and the surrounding areas to avoid causing cross-infection to the other areas, which belongs to the objective and unmodifiable requirements in epidemic prevention and control. Through the data collection of the surrounding greening distance of 39 locations in set $A$, 17 candidate points were excluded because of the non-compliant distance. Moreover, the final generated set ${A}_{w}$ contains 22 large-space public buildings, including 18 gymnasiums and 4 convention and exhibition centres.

The "Shili" factor evaluation criteria can be considered non-absolute indicators within a reasonable range. It is a roughly understanding that the larger or smaller the numerical value of certain criteria would be more conducive to the construction of makeshift hospitals. First and foremost, objective quantification methods for each criterion need to be defined.

Rescue efficiency will be affected by road, rail and air transportation, so for location accessibility, we specifically use road accessibility, air accessibility and rail accessibility to express alternative location accessibility. First, urban roads are the most critical transportation facilities in the city, and their accessibility index can reflect the convenience of the target facilities. In this paper, each candidate facility in set ${A}_{w}$ is used as the starting point to calculate the area covered by a fifteen-minute drive along the city road. Then, the number of residential settlements that each alternative facility in the fifteen-minute can cover counted through the visualization of isochronous circle analysis. In addition, as an efficient and large-capacity cross-regional transportation mode, the convenience of air and rail will have a significant impact on the arrival of medical aid and supplies, whose accessibility is mainly affected by factors such as the service capacity of the airport/train station, the distance between the alternative locations and the airport/train station and so on. Therefore, the air and railway accessibility index integrate service capacity and distance into the same index by adopting the potential model method. In this formula, the traffic accessibility index is proportional to the service capacity of airport/train station and inversely proportional to the distance. The formula is:

$${G}_{i}=\stackrel{n}{\underset{j=1}{\sum \left(\frac{{M}_{j}}{{D}_{ij}}\right)}}$$

${G}_{i}$ is the aviation accessibility index of candidate point $i$; ${M}_{j}$ is the service capacity of the airport; ${D}_{ij}$ is the actual distance from the candidate point $i$ to the airport $j$.

$${H}_{i}=\stackrel{n}{\underset{k=1}{\sum \left(\frac{{M}_{k}}{{D}_{ik}}\right)}}$$

${H}_{i}$ is the railway accessibility index of candidate point $i$; ${M}_{k}$ is the service capacity of the railway station; ${D}_{ik}$ is the actual distance from candidate point $i$ to the station j.

The distance from the location to the designated hospital: the actual distance from the candidate location to its nearest infectious disease hospital or third-grade class-A hospital. The formula is:

$$min{ L}_{ih}$$

$i$ is the candidate point; $h$ is the designated hospital that can provide services; ${L}_{ih}$ is the actual distance from the candidate point $i$ to the designated hospital $h$.

The scale of the site space: the available indoor area of the candidate point is divided by the specified per capita area. The number of temporary beds available of alternative locations can be equivalent to the size of the location.

$${C}_{i}={S}_{i}/A$$

${C}_{i}$ is the location space scale of the candidate point; ${S}_{i}$ is the available indoor area of the candidate point; $A$ is the specified per capita area.

According to the above evaluation criteria, the evaluation results of the "shili" factor are shown in Suppl Appendix 1, and the weight of each index calculated by the entropy weight method is shown in Table 3. At this time, we calculate the closeness ${E}_{i}$ of the candidate set ${A}_{w}$ to the ideal solution and arrange them in descending order according to the closeness ${E}_{i}$ to obtain the analytical results shown in Table 4. Finally, the top 50% of the rankings are taken to form the candidate set ${A}_{s}$ for selecting by "Renli" factors.

**Table 3:** "Shili" evaluation index weight.

	Accessibility			Distance to hospital	Space scale
	Air accessibility	Rail accessibility	Road accessibility		
Weight	0.06	0.08	0.17	0.02	0.67
Rank	4	3	2	5	1

**Table 4:** Screening results of "Shili" factor evaluation index.

Alternative facilities	${E}_{i}$	Rank
Chongming Gymnasium	0.027	21
Baoshan Gymnasium	0.046	15
New Jiading Gymnasium	0.045	18
Shanghai Sports Palace	0.155	10
Jing'an Sports Center	0.202	4
Yuanshen Gymnasium	0.157	9
Luwan Gymnasium	0.213	3
Shanghai Wanti Gymnasium	0.190	6
Minhang Gymnasium	0.103	12
Songjiang Gymnasium	0.047	14
Baogang Gymnasium	0.049	13
Jiading Gymnasium	0.042	19
Jiangwan Sports Center	0.169	7
Huangxing Sports Park	0.158	8
Caolu Sports Center	0.046	16
Dongfang Sports Center	0.138	11
Fengxian Gymnasium	0.032	20
Jinshan Gymnasium	0.015	22
Shanghai Automobile Exhibition Center	0.045	17
National Convention and Exhibition Center (Shanghai)	0.808	1
Shanghai New International Expo Centre	0.296	2
Shanghai International Sourcing Convention and Exhibition Center	0.199	5

The evaluation criteria for "Renli" factors are difficult to express clearly with specific mathematical formulas and mainly rely on the experience of decision-makers to make subjective judgments. The DMs first determined the weight of the evaluation index of the "renli" criteria according to the BWM method, as shown in Table 5. In addition, this paper uses the "renli" criteria to evaluate the facilities in the alternative set ${A}_{s}$ with the linguistic terms in Table 1, and obtain the initial evaluation value, as shown in suppl Appendix 2. Subsequently, the TOPSIS method was used to calculate the closeness $\widetilde{{E}_{\widetilde{i}}}$ of the ideal solution of the set ${A}_{s}$, and the alternative facilities were ranked in descending order (Table 6) to obtain the location selection order for the construction of Fangcang shelter hospitals. The results showed that the National Convention and Exhibition Center should be preferred as the Fangcang shelter hospital.

**Table 5:** "Renli" evaluation index weight.

	Operability	Post-pandemic building recovery	Operational effect	Implementation efficiency
Weight	0.57	0.06	0.14	0.23
Rank	1	4	3	2

**Table 6:** Screening results of "Renli" factor evaluation index.

Alternative facilities	Closeness	Rank
National Convention and Exhibition Center (Shanghai)	0.949398156	1
Shanghai New International Expo Centre	0.833905844	3
Luwan Gymnasium	0.768748131	5
Jing'an Sports Center	0.785615886	4
Shanghai International Sourcing Convention and Exhibition Center	0.860039008	2
Shanghai Wanti Gymnasium	0.722281782	9
Jiangwan Sports Center	0.765861257	6
Huangxing Sports Park	0.117826508	11
Yuanshen Gymnasium	0.750361649	7
Shanghai Sports Palace	0.710431418	10
Dongfang Sports Center	0.745970649	8

## Results and discussions

After screening through the location selection decision-making framework, the National Convention and Exhibition Center has the advantage of being located at a central point that can be reached by air or roadways, with many indoor and outdoor empty areas, far from residential areas, extensive infrastructure, and so on. Therefore, transforming the National Convention and Exhibition Center into a Fangcang shelter hospital by equipping it with the necessary tools and health professionals to receive patients also meets the expectations of government administrators and DMs. Relevant studies have shown that the Fangcang shelter hospitals built during the epidemic in Shanghai, especially the transformation of the National Convention and Exhibition Center, played an essential role in curbing the epidemic^57^.

Following National Convention and Exhibition Center, many exhibition halls and gyms with large areas, accessible transportation, and being away from crowded districts have also been selected as the appropriate location for Fangcang shelter hospitals. During the Shanghai epidemic, they were successfully converted into Fangcang shelter hospitals to receive patients. Overall, after the three-level screening of "Wuli'"–"Shili"–"Renli", the final ranking order of the alternative locations suitable for the establishment of Fangcang shelter hospitals was obtained in this case. Compared with the actual construction and use during the epidemic, the top seven in the final ranking have experience being used as emergency medical facilities, of which the top six are used as the Fangcang shelter hospitals. In particular, the ranking 1 National Convention and Exhibition Center (Shanghai) was transformed into the largest Fangcang shelter hospital in Shanghai, which played an essential role in stabilizing the epidemic in Shanghai. Since then, with the decline of the ranking, the seventh-ranked Yuanshen Sports Center was used as a transfer station for recovered patients to return to the community during the epidemic. Besides, the last four alternative facilities were not used due to their location, area, and other deficiencies.

### Comparative and sensitivity analysis

To validate the effectiveness and feasibility of the proposed method for evaluating alternative locations to set up Fangcang Shelter hospitals, sensitivity analysis was directed to check the robustness of the results. We manipulated the criteria weights and created ten cases consisting of extreme cases. The weights of the criteria in the "Wuli" factor were not adjusted because of their absoluteness. Ten groups of weight combinations (Tables 7 and 8) were created for the "Shili" and "Renli" factors, such as selecting only the most essential criterion and considering the weights of other criteria the same and vice versa.

**Table 7:** Weights of criteria determined for sensitivity analysis—"Shili".

Cases	Accessibility			Distance to hospital	Space scale
	Air accessibility	Rail accessibility	Road accessibility		
1	0.04	0.04	0.44	0.04	0.44
2	0.04	0.24	0.24	0.24	0.24
3	0.08	0.08	0.28	0.28	0.28
4	0.07	0.07	0.36	0.07	0.43
5	0.13	0.17	0.25	0.12	0.33
6	0.13	0.13	0.13	0.13	0.48
7	0.03	0.25	0.26	0.29	0.17
8	0.11	0.16	0.23	0.28	0.22
9	0.27	0.09	0.47	0.11	0.06
10	0.12	0.12	0.52	0.12	0.12

**Table 8:** Weights of criteria determined for sensitivity analysis—"Renli".

Cases	Operability	Post-pandemic building recovery	Operational effect	Implementation efficiency
1	0.61	0.13	0.13	0.13
2	0.43	0.07	0.07	0.43
3	0.25	0.25	0.25	0.25
4	0.31	0.07	0.31	0.31
5	0.34	0.19	0.25	0.22
6	0.29	0.29	0.13	0.29
7	0.23	0.06	0.14	0.57
8	0.43	0.07	0.43	0.07
9	0.52	0.06	0.19	0.23
10	0.57	0.06	0.06	0.31

**Figure 6:**
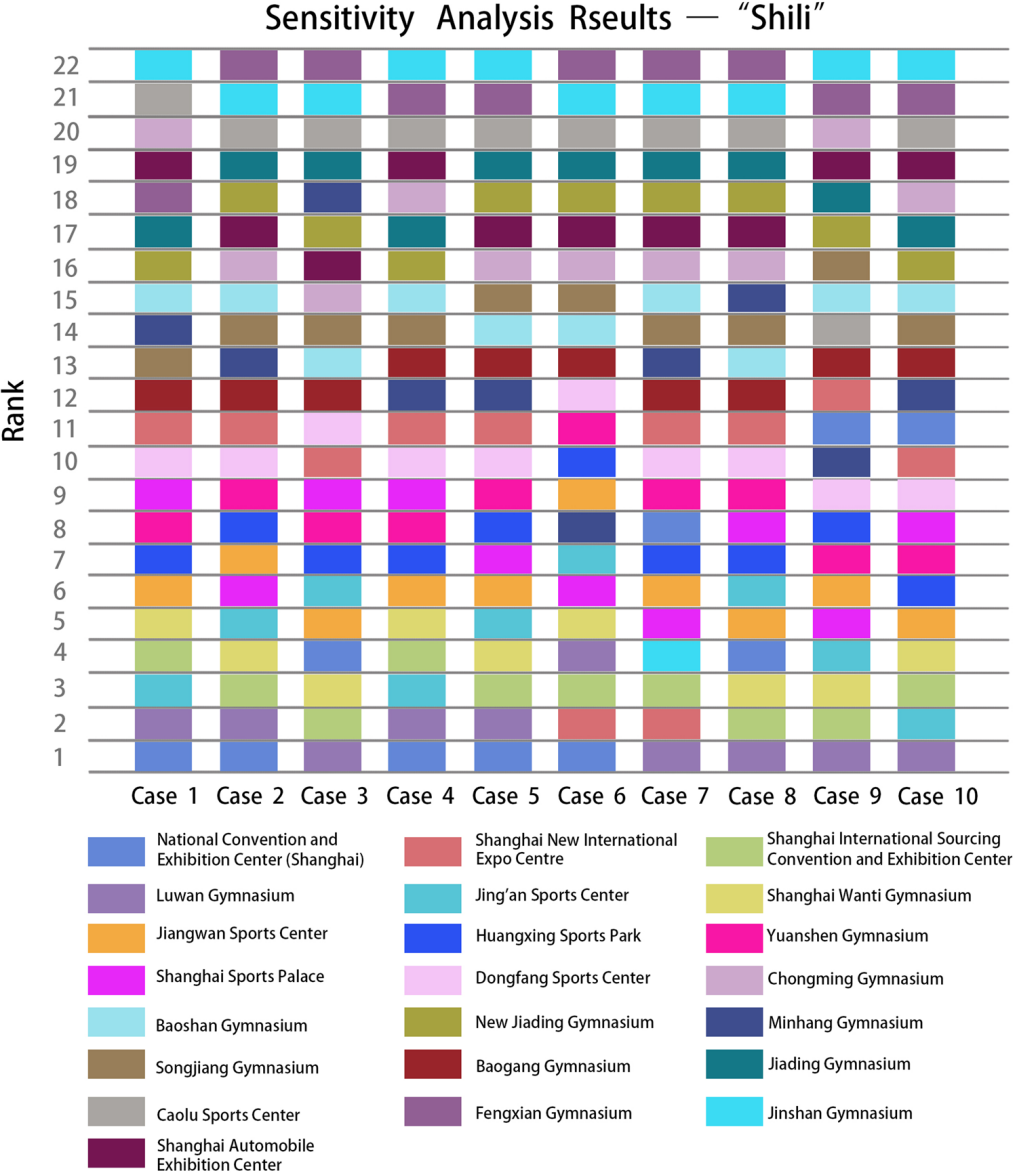
Rank of alternative facilities with respect to different criteria weights—"Shili".

**Figure 7:**
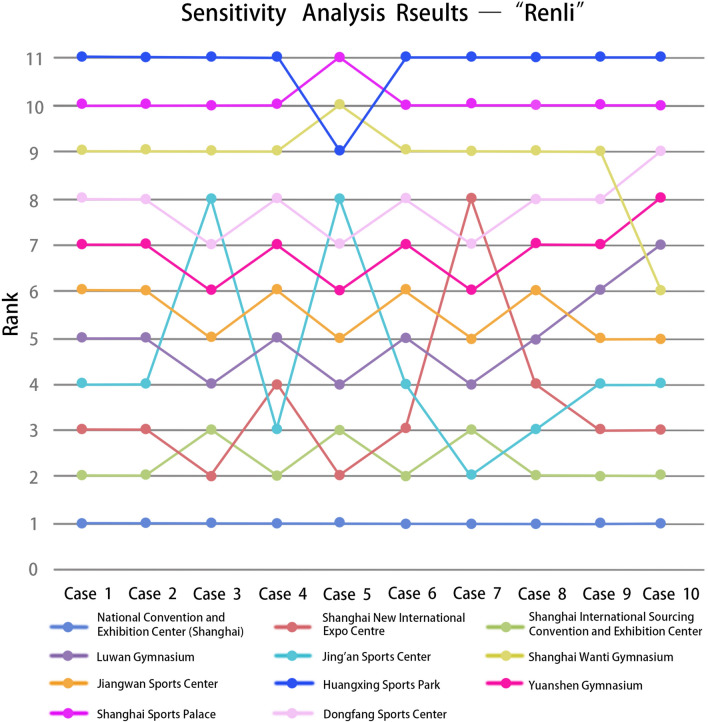
Rank of alternative facilities with respect to different criteria weights—"Renli”.

The sensitivity analysis results in the "Shili" stage (Fig. 6) showed that the ranking fluctuations of each alternative facility were between 1–2 rankings. The selection of the most and least suitable facilities for transformation into Fangcang shelter hospitals remained unchanged. Among the test cases, eight groups of the top 11 alternative facilities are consistent, and only one of the remaining two groups of cases is different from the previous results. In the sensitivity analysis of "Renli" factors, alternatives with the same evaluation results will be ranked according to the "Shili" factor. Once the 11 cases of “Renli” factors in sensitivity analysis were analyzed, it was seen that the top three and the last three are consistent, as the National Convention and Exhibition Center (Shanghai) ranked as the first while Huangxing Sports Park was ranked as the last (Fig. 7). Except for Jing'an Sports Center, the ranking positions of other cases fluctuated between 1 and 2, which had little influence on the final screening results.

On the other hand, the changes in rankings showed the sensitivity of the ranking procedure of the framework considering the criteria weights. In particular, the ranking was changed significantly when the weight of operability was the highest, which meant the results were sensitive to operability. Overall, as seen in case 1, 2, and 10, when the weight of the operational effect is the lowest, the final ranking fluctuation is relatively stable. Moreover, as seen in case 4, 5, and 7, the ranking fluctuation is significant when the weight of post-pandemic building recovery is low.

In a word, sensitivity analysis showed that the framework produced robust and valid results. Consequently, the comparative analysis showed that the WSR methodology-based MCDM framework that integrates EWB, BWM, and interval type‐2 fuzzy TOPSIS method is efficient and consistent with actual usage.

## Conclusions and limitations

Emergency medical facilities are essential to alleviate local excessive medical demand load after major emergencies. To reduce medical evacuation time and improve the possibility of survival, efficient and scientific planning of the location of emergency medical facilities is a significant way to improve the efficiency of first aid, especially for urban areas with high-density populations. However, previous studies I have not fully considered the difference between "issue" and "problem" factors in the influencing factors of location selection. It often comprehensively considers all the factors affecting the location selection at one time by using one method to solve all factors, which challenging in taking into account the impact of factors with different attributes in different stages on the final location selection results.

Guided by the WSR methodology, this study constructed a three-level screening method for emergency facility location selection by distinguishing between "issue" and "problem" factors. Specifically, this study used different methods to screen and judge each "Wuli", "Shili" and "Renli" factor. The first step is to directly screen out the candidate points that do not meet the objective requirements of "Wuli" factors. For example, we can use GIS to analyze the location of emergency medical facilities because of the spatial nature of the potential criteria. The second step is to assign values to determine the index weight of the "Shili" factor index composed of objective data with the entropy weight method to secondary screen the alternative facilities with the TOPSIS method. The third step is to determine the "Renli" factor index weights constituted by the subjective evaluation with the BWM method to finalize the selection of alternative facilities with the TOPSIS method. Finally, a case study was presented in detail to indicate the application of the proposed location selection framework, and the final screening results can prove that it can well serve the actual location selection needs. The sensitivity analysis validated the robustness of the method. To sum up, the validation of the effectiveness, feasibility, and robustness of the proposed method is proved. This framework can be easily applied to other cities to meet the location needs of emergency medical facilities.

The current study can be extended in several directions. First, WSR methodology-based multi-criteria decision‐making framework can be employed to determine the most suitable locations for emergency facilities. Second, the suitable criteria to be used can be evaluated by using the interval type‐2 fuzzy technique. Finally, the interrelationships among the factors that affect the location selection can be analyzed using EWM-TOPSIS or BWM-TOPSIS.

Even though this study provides a decision-making framework for emergency facility location selection in response to major emergencies through the proposal of the scientific three-level screening method of "Wuli'"–"Shili"–"Renli" and the verification of practical application cases, certain limitations should be considered in evaluating the conclusions of this research. Based on the "hypothesis of rational man", the established location selection framework assumes that the members of DMs can make rational decisions in line with the actual situation under the control of relatively complete information. However, decision-makers often lack relevant practical experience and relatively limited information obtained in a short time, which may deviate the decision-making process and result. Therefore, it is not only necessary to strengthen the relevant training of professionals in the future to enhance the understanding of various emergencies and the information of the city but also to establish an information management platform for large urban public buildings and to enter relevant data information in advance to shorten the acquisition time of information in emergencies. Apart from these, it is better to optimize further the decision-making process of the "Shili" factor and "Renli" factor and seek a more simplified and efficient calculation and screening method.

### Supplementary Information

Supplementary Information 1.

Supplementary Information 2.

## Data Availability

All data generated and analyzed during this study are included in this published article.
