# Supplementary material for: Research on the location decision-making method of emergency medical facilities based on WSR
Source: Sci Rep. 2023 Oct 21;13:18011. doi: 10.1038/s41598-023-44209-0 (PMC10590399; doi:10.1038/s41598-023-44209-0)
Supplement: Supplementary file 1 — Supplementary Information 1. [file 41598_2023_44209_MOESM1_ESM.docx]

| **Appendix 1** "Shili" evaluation index data | | | | | |
| --- | --- | --- | --- | --- | --- |
| **Alternative facilities** | **Accessibility** | | | **Distance to hospital (km)** | **Space scale (bed)** |
|  | **Air accessibility** | **Rail accessibility** | **Road accessibility** |  |  |
| Chongming Gymnasium | 3451.75 | 6662.61 | 69 | 1.4 | 300 |
| Baoshan Gymnasium | 8242.82 | 22757.47 | 429 | 2.8 | 400 |
| New Jiading Gymnasium | 7756.46 | 24824.60 | 452 | 7.2 | 400 |
| Shanghai Sports Palace | 16182.61 | 57837.32 | 2135 | 4.2 | 300 |
| Jing'an Sports Center | 11155.40 | 49580.08 | 3181 | 7.5 | 800 |
| Yuanshen Gymnasium | 11800.42 | 38262.55 | 2342 | 3.4 | 300 |
| Luwan Gymnasium | 14584.04 | 65807.42 | 3346 | 1 | 400 |
| Shanghai Wanti Gymnasium | 15810.81 | 62689.90 | 2807 | 3.4 | 800 |
| Minhang Gymnasium | 20964.19 | 66998.90 | 329 | 9 | 500 |
| Songjiang Gymnasium | 7603.36 | 23950.60 | 482 | 3 | 400 |
| Baogang Gymnasium | 8019.47 | 21537.92 | 471 | 0.42 | 500 |
| Jiading Gymnasium | 7569.21 | 24071.51 | 404 | 7.3 | 400 |
| Jiangwan Sports Center | 10194.21 | 33821.49 | 2604 | 1.8 | 500 |
| Huangxing Sports Park | 10504.86 | 34708.34 | 2397 | 1.8 | 400 |
| Caolu Sports Center | 11883.66 | 20617.55 | 134 | 10.8 | 200 |
| Dongfang Sports Center | 2291.49 | 42495.19 | 1962 | 6.7 | 500 |
| Fengxian Gymnasium | 7589.37 | 19023.90 | 365 | 26.2 | 300 |
| Jinshan Gymnasium | 5027.21 | 14552.48 | 99 | 18 | 300 |
| Shanghai Automobile Exhibition Center | 7553.28 | 27252.47 | 150 | 5.3 | 1500 |
| National Convention and Exhibition Center (Shanghai) | 16776.13 | 109125.24 | 259 | 11.1 | 50000 |
| Shanghai New International Expo Centre | 11769.00 | 31462.17 | 872 | 4.5 | 15000 |
| Shanghai International Sourcing Convention and Exhibition Center | 19941.60 | 60664.76 | 2754 | 2.2 | 2300 |
