# Supplementary material for: Research on the location decision-making method of emergency medical facilities based on WSR
Source: Sci Rep. 2023 Oct 21;13:18011. doi: 10.1038/s41598-023-44209-0 (PMC10590399; doi:10.1038/s41598-023-44209-0)
Supplement: Supplementary file 2 — Supplementary Information 2. [file 41598_2023_44209_MOESM2_ESM.docx]

| Appendix 2 "Renli" evaluation index data | | | | |
| --- | --- | --- | --- | --- |
| Alternative facilities | **Operability** | **Post-pandemic building recovery** | **Operational effect** | **Implementation efficiency** |
| National Convention and Exhibition Center (Shanghai) | ((9,10,10,10;1,1),(9.5,10,10,10;0.9,0.9)) | ((8,9,10,10;1,1),(9,9,9,10;0.9,0.9)) | ((7,9,9,10;1,1),(8,9,9,9.5;0.9,0.9)) | ((8,9,10,10;1,1),(9,9,9,10;0.9,0.9)) |
| Shanghai New International Expo Centre | ((8,9,10,10;1,1),(9,9,9,10;0.9,0.9)) | ((7,9,9,10;1,1),(8,9,9,9.5;0.9,0.9)) | ((5,7,7,9;1,1),(6,7,7,8;0.9,0.9)) | ((8,9,10,10;1,1),(9,9,9,10;0.9,0.9)) |
| Luwan Gymnasium | ((7,9,9,10;1,1),(8,9,9,9.5;0.9,0.9)) | ((1,3,3,5;1,1),(2,3,3,4;0.9,0.9)) | ((7,9,9,10;1,1),(8,9,9,9.5;0.9,0.9)) | ((9,10,10,10;1,1),(9.5,10,10,10;0.9,0.9)) |
| Jing'an Sports Center | ((7,9,9,10;1,1),(8,9,9,9.5;0.9,0.9)) | ((3,5,5,7;1,1),(4,5,5,6;0.9,0.9)) | ((7,9,9,10;1,1),(8,9,9,9.5;0.9,0.9)) | ((9,10,10,10;1,1),(9.5,10,10,10;0.9,0.9)) |
| Shanghai International Sourcing Convention and Exhibition Center | ((8,9,10,10;1,1),(9,9,9,10;0.9,0.9)) | ((8,9,10,10;1,1),(9,9,9,10;0.9,0.9)) | ((7,9,9,10;1,1),(8,9,9,9.5;0.9,0.9)) | ((8,9,10,10;1,1),(9,9,9,10;0.9,0.9)) |
| Shanghai Wanti Gymnasium | ((7,9,9,10;1,1),(8,9,9,9.5;0.9,0.9)) | ((3,5,5,7;1,1),(4,5,5,6;0.9,0.9)) | ((3,5,5,7;1,1),(4,5,5,6;0.9,0.9)) | ((8,9,10,10;1,1),(9,9,9,10;0.9,0.9)) |
| Jiangwan Sports Center | ((7,9,9,10;1,1),(8,9,9,9.5;0.9,0.9)) | ((3,5,5,7;1,1),(4,5,5,6;0.9,0.9)) | ((5,7,7,9;1,1),(6,7,7,8;0.9,0.9)) | ((9,10,10,10;1,1),(9.5,10,10,10;0.9,0.9)) |
| Huangxing Sports Park | ((3,5,5,7;1,1),(4,5,5,6;0.9,0.9)) | ((8,9,10,10;1,1),(9,9,9,10;0.9,0.9)) | ((1,3,3,5;1,1),(2,3,3,4;0.9,0.9)) | ((8,9,10,10;1,1),(9,9,9,10;0.9,0.9)) |
| Yuanshen Gymnasium | ((7,9,9,10;1,1),(8,9,9,9.5;0.9,0.9)) | ((1,3,3,5;1,1),(2,3,3,4;0.9,0.9)) | ((5,7,7,9;1,1),(6,7,7,8;0.9,0.9)) | ((9,10,10,10;1,1),(9.5,10,10,10;0.9,0.9)) |
| Shanghai Sports Palace | ((7,9,9,10;1,1),(8,9,9,9.5;0.9,0.9)) | ((1,3,3,5;1,1),(2,3,3,4;0.9,0.9)) | ((3,5,5,7;1,1),(4,5,5,6;0.9,0.9)) | ((8,9,10,10;1,1),(9,9,9,10;0.9,0.9)) |
| Dongfang Sports Center | ((7,9,9,10;1,1),(8,9,9,9.5;0.9,0.9)) | ((1,3,3,5;1,1),(2,3,3,4;0.9,0.9)) | ((5,7,7,9;1,1),(6,7,7,8;0.9,0.9)) | ((8,9,10,10;1,1),(9,9,9,10;0.9,0.9)) |
